# Comparative Analysis of Transposable Elements in the Genomes of *Citrus* and *Citrus*-Related Genera

**DOI:** 10.3390/plants13172462

**Published:** 2024-09-03

**Authors:** Yilei Wu, Fusheng Wang, Keliang Lyu, Renyi Liu

**Affiliations:** 1 College of Life Sciences, Fujian Agriculture and Forestry University, Fuzhou 350002, China; 2 Center for Agroforestry Mega Data Science, Haixia Institute of Science and Technology, Fujian Agriculture and Forestry University, Fuzhou 350002, China; 3 National Citrus Engineering Research Center, Citrus Research Institute, Southwest University, Chongqing 400712, China

**Keywords:** *Citrus*, transposable elements (TEs), pan-genome TE library, genome evolution, bitterness

## Abstract

Transposable elements (TEs) significantly contribute to the evolution and diversity of plant genomes. In this study, we explored the roles of TEs in the genomes of *Citrus* and *Citrus*-related genera by constructing a pan-genome TE library from 20 published genomes of *Citrus* and *Citrus*-related accessions. Our results revealed an increase in TE content and the number of TE types compared to the original annotations, as well as a decrease in the content of unclassified TEs. The average length of TEs per assembly was approximately 194.23 Mb, representing 41.76% (*Murraya paniculata*) to 64.76% (*Citrus gilletiana*) of the genomes, with a mean value of 56.95%. A significant positive correlation was found between genome size and both the number of TE types and TE content. Consistent with the difference in mean whole-genome size (39.83 Mb) between *Citrus* and *Citrus*-related genera, *Citrus* genomes contained an average of 34.36 Mb more TE sequences than *Citrus*-related genomes. Analysis of the estimated insertion time and half-life of long terminal repeat retrotransposons (LTR-RTs) suggested that TE removal was not the primary factor contributing to the differences among genomes. These findings collectively indicate that TEs are the primary determinants of genome size and play a major role in shaping genome structures. Principal coordinate analysis (PCoA) of Gene Ontology (GO) and Kyoto Encyclopedia of Genes and Genomes (KEGG) identifiers revealed that the fragmented TEs were predominantly derived from ancestral genomes, while intact TEs were crucial in the recent evolutionary diversification of *Citrus*. Moreover, the presence or absence of intact TEs near the AdhE superfamily was closely associated with the bitterness trait in the *Citrus* species. Overall, this study enhances TE annotation in *Citrus* and *Citrus*-related genomes and provides valuable data for future genetic breeding and agronomic trait research in *Citrus*.

## 1. Introduction

Transposable elements (TEs), mobile and repetitive nucleic acid sequences dispersed throughout the genomes of most eukaryotes, play critical roles in genome structure, evolution, and gene function differentiation [1,2]. TEs are major components of plant genomes, constituting approximately 10%, 35%, 40%, and 85% of the whole genome of *Arabidopsis thaliana* [3], *Oryza sativa* [4], *Citrus* L. [5], and *Zea mays* [6], respectively. Based on their intermediate sequence and transposition mechanism, TEs can be classified into two main classes: Class I TE elements (retrotransposons) consisting of long terminal repeats (LTRs), long interspersed nuclear elements (LINEs), and short interspersed nuclear elements (SINEs), which mobilize through a ‘copy-and-paste’ mechanism via an RNA intermediate [7], and Class Ⅱ TE elements (DNA transposons) consisting of terminal inverted repeats (TIRs) and Helitrons, which mobilize through ‘cut-and-paste’ or ‘peel-and-paste’ replicative mechanisms [7]. Although Cryptons and Mavericks also belong to Class II TEs, they have not yet been identified in plants [7,8,9].

TEs are recognized as insertional mutagens and major drivers of genome evolution, significantly contributing to the rapid adaptation and domestication of almost all plants [2,10]. They are prevalent in both gene-poor, repeat-rich heterochromatic regions as well as gene-rich regions of genomes [11,12]. Accumulating evidence has revealed that TE abundance not only varies across different genera but also within the same species [13]. However, the ultimate causes of such dramatic divergences in TE abundance in genomes remain unclear.

Since their first discovery in maize in 1950 [14], TEs have been reported to disrupt genes and functional elements, generate novel genes, introduce new genetic functions and ectopic regulatory sequences, and affect gene expression [15,16,17,18]. The relationship between the TE insertions and the fruit color of Japanese plum and apple has recently been explored [19,20]. In tomatoes, genome-wide association studies have identified 40 TE insertion polymorphisms highly associated with agronomic traits or secondary metabolites [21]. All these findings highlighted the significance of TEs in genomics, breeding, hybridization, and phylogeny. However, most studies have been based on a single reference genome due to the limited availability of genome assemblies and the challenges associated with repetitive sequence assembly and annotation [22]. Additionally, misinterpretations arising from different TE annotation methods undermine the reliability of TE comparisons. A pan-genome TE library can merge TE libraries from multiple accessions, enabling consistent reannotation of each genome, homology-based annotation of fragmented elements, and standardized TE family names across all analyzed genomes [22,23]. Exploring TEs in a pan-genome context may be an effective approach to combine high-quality TE annotations and characterize TE variabilities among different genomes.

*Citrus* is among the most extensively cultivated and economically important fruit crops globally. However, various factors, such as sprout mutations, clonal propagation via nucellar embryony, inter- and intraspecific crosses, grafting, and clonal selection, have contributed to the complex genealogy of *Citrus*, significantly impacting genome structures [5,24,25]. While the importance of TEs in *Citrus* genomes was largely underestimated before the publication of the sweet orange draft genome, it is now known that approximately half of *Citrus* genomes consist of highly repetitive regions populated by TEs [5,26]. The insertion of a *Copia* retrotransposon near the *Ruby* gene and controls its expression and the anthocyanin accumulation under low temperature in blood orange was reported by Butelli et al. [27]. Hu et al. [28] revealed that the insertion of a miniature inverted-repeat transposable element (*MITE*) in the promoter region is important for self-incompatibility in *Citrus*, while Wang et al. [29] discovered that a TE insertion in the *CitRWP* gene is responsible for *Citrus* apomixis.

Research has also identified a correlation between TE insertions in acid transporter genes or their regulatory regions [27,30]. Accurate annotation of TEs is vital for genomic research and understanding the evolutionary processes in *Citrus*. For instance, pan-genome analyses of *Citrus* and *Citrus*-related genera have revealed that 54–77% of presence and absence variations (PAVs) are derived from TEs [13], highlighting their genomic importance. However, most previous studies have relied on a single genome or an unannotated pan-genome TE library, hindering comparative TE analysis and functional assessment of specific TEs across *Citrus* and related genera.

Twenty complete genome assemblies of *Citrus* and *Citrus*-related accessions are available in the Citrus Pan-genome to Breeding Database (CPBD, http://citrus.hzau.edu.cn, accessed on 2 October 2023) [31], providing a valuable resource for the systematic identification, annotation, and comparison of TEs. In the current study, these 20 high-quality genomes were used for the comprehensive identification of TEs and the construction of a pan-genome TE library. The abundance of TEs in different accessions and their role in genome structure and evolution were investigated. Gene comparisons within the *Citrus* genomes suggested that the presence or absence of intact TEs may have significant implications for *Citrus* bitterness. The results obtained in this study will be instrumental in mapping the landscapes of TEs in *Citrus* and *Citrus*-related genera, thereby offering a foundation for understanding the evolution and subspeciation of *Citrus* species.

## 2. Results

### 2.1. Construction of the Pan-Genome TE Library

Transposable elements (TEs) were identified in the genomes of 20 *Citrus* and *Citrus*-related genera, including 14 *Citrus* and six *Citrus*-related accessions (Table 1). A total of 69,338 consensus TE sequences were identified using EDTA [32], with each genome yielding between 1698 and 4206 consensus TE sequences. A pan-genome TE library, consisting of 21,680 non-redundant TE sequences, was constructed using an iterative approach with the consensus TEs of 20 *Citrus* and *Citrus*-related genera. Within this library, 8012 retrotransposons (Class I) and 13 668 DNA transposons (Class II) were identified. The number of *Copia* LTR-RTs (4072) was greater than that of *Gypsy* LTR-RTs (3427) (Table 2).

### 2.2. Summary of TEs in Reannotated Genomes

**Figure 1:**
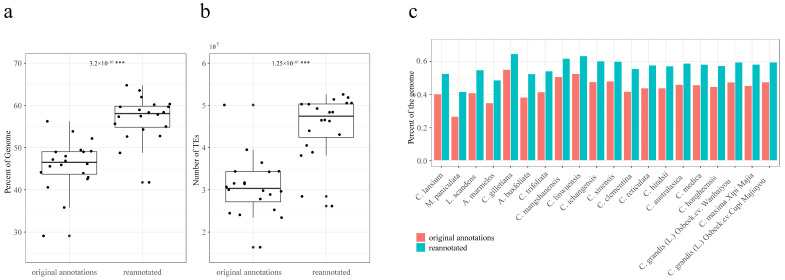
TE re-annotations using the pan-genome TE library. (**a**) Comparisons of the TE proportions between originally annotated and re-annotated genomes. (**b**) Comparisons of TE numbers between originally annotated and re-annotated genomes. (**c**) TE contents of originally annotated and re-annotated genomes. *p* values less than 0.001 are indicated with ***.

Whole-genome TE annotation was performed using the pan-genome TE library. The total length and number of annotated TEs significantly increased compared to the original annotations (Figure 1a,b). Average TE length per assembly ranged from 90.8 to 271.0 Mb (accounting for 41.8–64.8% of the total assembly), with an average length of 194.23 Mb (56.95% of the total assemblies), greater than that of the original annotations (Figure 1c). *Citrus*-related genera exhibited high TE content variation, ranging from 41.76% to 64.76%. In contrast, all *Citrus* accessions showed similar TE content, ranging from 54.29% to 63.54% (Figure 1c).

The TE annotation results for the 20 genomes indicated that the average proportions of LTR-RTs and TIRs were 28.97% (ranging from 17.9% to 34.6%) and 23.16% (ranging from 14.25% to 34.1%), respectively, both of which exceeded the original annotations (ranging from 12.5% to 32.2%, Supplementary Table S1, Supplementary Figure S1). The LTR-RTs constituted a higher proportion of TE content across all genomes. However, the proportion of LTR-RTs in *C. sinensis* and *M. paniculata* was slightly lower than that of TIRs (0.4% and 1.8%, respectively), while the proportion of LTR-RTs in *C. linwuensis* and *C. mangshanensis* was substantially lower (4% and 9.4%, respectively). Additionally, the number and percentage of *Gypsy* LTR-RTs exceeded those of *Copia* LTR-RTs across all accessions (Supplementary Table S1).

*MITE* (1.12%), P elements (*P*, 0.01%), DIRS retrotransposons (*DIRS_YR*, 0.01%), LINE I elements (*I_LINE*, 0.01%), LINE-1 elements (*L1_LINE*, 0.14%), Penelope retrotransposons (*Penelope*, 0.14%), and *Helitrons* (0.60%) were identified in the re-annotation dataset but were absent in original genome annotations. Additionally, *Polinton* (also known as *Maverick*) was identified in *C. hongheensis* (Supplementary Table S1). Interestingly, the number of unclassed TEs (reclassified as Repeat_region) decreased (Supplementary Figure S2), suggesting that the observed differences in unclassified TEs in the original annotations were due to annotation artifacts.

To determine the distribution of TEs in *Citrus* and *Citrus*-related genera, we selected six chromosome-level assemblies (*C. hindsii*, *C. sinensis*, *C. grandis* (L.) Osbeck. cv. Cupi Majiayou, *C. grandis* (L.) Osbeck. cv. Wanbaiyou, *C. trifoliata*, and *A. buxifolia*) for further analysis. The chromosomal distribution of TEs was uneven, with fewer TEs at the chromosome ends (Supplementary Figure S3). Higher concentrations of TEs were detected near the middle of chromosomes in *A. buxifolia* (chr4, chr6, and chr8), *C. sinensis* (chr2), and *C. grandis* (L.) Osbeck. cv. Cupi Majiayou (chr3 and chr9).

### 2.3. TE Evolutionary History in Citrus and Citrus-Related Genera

**Figure 2:**
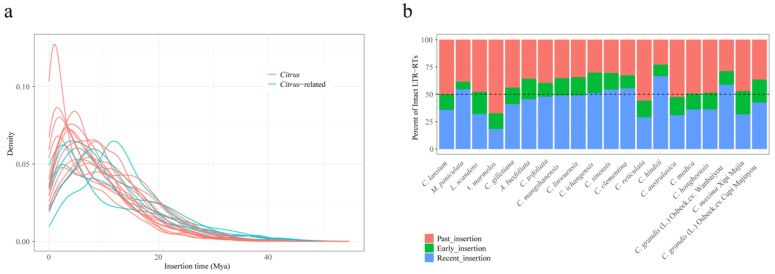
The insertion times of LTR retrotransposons (LTR-RTs). (**a**) Distributions of LTR-RT insertion times in each genome. Red lines and blue lines represent genomes in *Citrus* and *Citrus*-related genera, respectively. (**b**) The percentages for different types of LTR-RTs. Recent insertion represents the TE inserted at 0–6.84 Mya, early insertion represents the TE inserted at 6.84–10 Mya, and past insertion represents the TE inserted before 10 Mya.

To analyze the activity history of LTR-RTs within the *Citrus* and *Citrus*-related genera, distribution curve analysis of the insertion time of intact LTR-RTs for each subgenome was performed. Results showed that most LTR-RT insertions in *Citrus* were relatively recent (Figure 2a). In 17 of the 20 accessions, over 50% of LTR-RTs were inserted after the early divergence of *Citrus* around 10 million years ago (Mya) (Figure 2b). A substantial proportion of TE insertions were defined as recent (0–6.84 Mya) (Figure 2b). Results indicated that the burst times of LTR-RT insertions varied among the *Citrus* and *Citrus*-related genera, with 19 genomes exhibiting burst times more recent than the early divergence of *Citrus*, ranging from 0.99 Mya in *C. hindsii* to 11.86 Mya in *A. marmelos* (Table 1). These results suggest the occurrence of relatively independent LTR-RT insertions after speciation.

**Figure 3:**
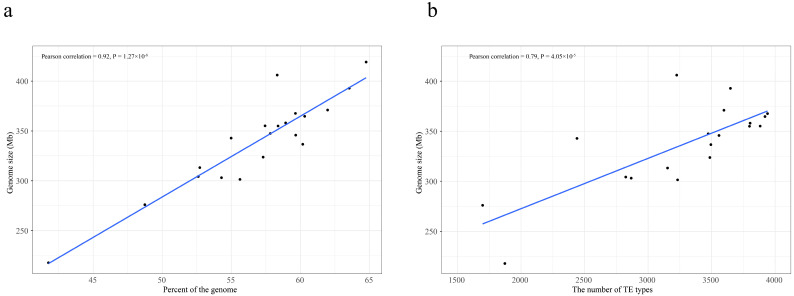
Correlation analysis between genome size and content of TEs (**a**) and between genome size and the number of TE types (**b**) in 20 genomes.

The impact of TE content on genome size was evaluated, revealing a significant positive correlation between the proportion of TEs and genome size, confirming that TEs are a major determinant of genome size in *Citrus* and *Citrus*-related genera (Pearson correlation coefficient: 0.92, *p* = 1.27 × 10^−8^, Figure 3a). The curated TE library generated by EDTA considered each consensus sequence as a TE family. Further correlation analysis indicated a significant positive correlation between the number of consensus TEs and genome size (Pearson correlation coefficient: 0.79, *p* = 4.05 × 10^−5^, Figure 3b), highlighting the influence of the number of TE families on genome size.

The half-life rate was used to evaluate the removal speed of TE sequences from the genomes. Results indicated that the median removal rate of LTR-RTs was 6.92 million years, ranging from 4.20 million years in *C. hindsii* to 9.51 million years in *A. marmelos* (Table 1). No significant correlations were found between the half-life rate and genome size among all accessions or just chromosome-level accessions. These findings suggest that the relatively high half-life rate (indicating low TE removal) had no obvious effect on genome size in *Citrus* and *Citrus*-related genera.

### 2.4. TE Variation in Citrus and Citrus-Related Genera

**Figure 4:**
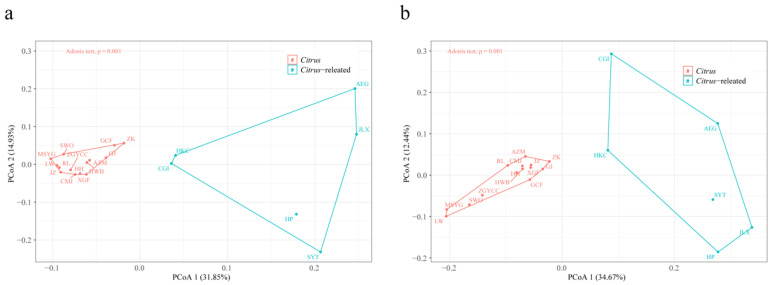
(**a**) Principal coordinate analysis (PCoA) analysis results based on the copy number of TE families across genomes. (**b**) PCoA plots based on the size of TE families across genomes. The closer the spatial distance of the sample, the more similar the sample. The statistical differences of PCoA were calculated by the Adonis test. *A. marmelos*, *A. buxfoliata*, *C. gilletiana*, *C. lansium*, *L. scandens*, *M. paniculate* (AEG, HKC, CGI, HP, SYT, JLX) were defined as *Citrus*-related accessions; *C. trifoliata*, *C. mangshanensis*, *C. linwuensis*, *C. ichangensis*, *C. sinensis*, *C. clementina*, *C. reticulata*, *C. hindsii*, *C. australasica*, *C. medica*, *C. hongheensis*, *C. grandis* (L.) Osbeck cv. Wanbaiyou, *C. maxima* Xipi Majia, *C. grandis* (L.) Osbeck cv. Cupi Majiayou (ZK, MSYG, LW, ZGYCC, SWO, GCF, JZ, GJ, AZM, RL, HH, HWB, XGF, CMJ) were defined as *Citrus* accessions.

The copy number and size of TE families exhibited variation across different genomes. Principal coordinate analysis (PCoA) based on these parameters revealed significant divergence between *Citrus* and *Citrus*-related genera (Adonis test *p* = 0.001, Figure 4a; Adonis test *p* = 0.001, Figure 4b). Wilcoxon tests (*p* < 0.05) identified highly variable families, with 1597 families differing in copy number and 2206 families differing in TE size between the genomes of *Citrus* and *Citrus*-related genera (Supplementary Tables S2 and S3). The *Citrus* genomes contained an average of 53.68 Mb (ranging from 43.39 to 63.61 Mb, accounting for 12.67% to 17.31% of the total genome) of highly variable TE sequences, while *Citrus*-related genera contained an average of 19.32 Mb (ranging from 12.69 to 26.83 Mb, accounting for 5.23% to 8.82% of the total genome). The net difference in TE sequences between *Citrus* and *Citrus*-related genera was 34.36 Mb, closely matching the mean difference in total genome size (39.83). LTR-RTs contributed 62.48% (21.47 Mb) of the total TE variation, with *Gypsy* elements showing the largest size difference (16.32 Mb more in *Citrus* genomes). Unclassified TIRs, *Mutator*, *hAT*, and *CACTA,* contributed 11.65% (4.01 Mb), 8.53% (2.93 Mb), and 5.48% (1.88 Mb) of the total TE variation, respectively. Other TE variations between *Citrus* and *Citrus*-related genera genomes accounted for less than 5%. These findings suggest that TE insertions are a predominant force in shaping the genome structures of *Citrus* and *Citrus*-related genera.

### 2.5. Important Implications of TEs for Citrus Bitterness

TE insertions represent a considerable source of intraspecific variation. Based on screening the overlaps between genomic features and both structurally intact and fragmented TEs, the average number of genes overlapping with intact TEs per assembly was 949 (539–2254), accounting for 3.28% (1.90–6.92%) of the genome. The number of genes overlapping with fragmented TEs per assembly was larger, accounting for 47.18% (32.88–54.74%) of the genome annotation for each accession.

**Figure 5:**
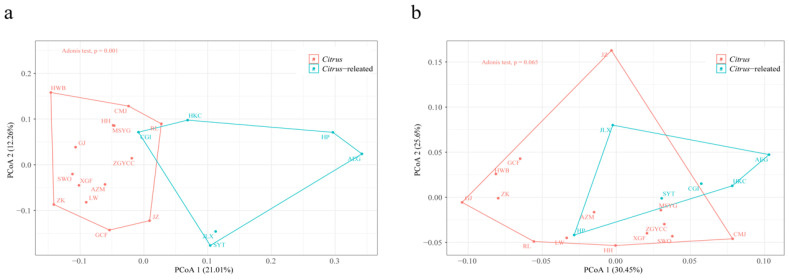
PCoA plots for KEGG identifier distribution of genes that overlapped with intact TEs (**a**) and KEGG identifier distribution of genes that overlapped with fragmented TEs (**b**) between the *Citrus* and *Citrus*-related accessions. The closer the spatial distance of the sample, the more similar the KEGG identifier composition of the sample. The statistical differences of PCoA were calculated by an Adonis test. *A. marmelos, A. buxfoliata*, *C. gilletiana*, *C. lansium*, *L. scandens*, *M. paniculate* (AEG, HKC, CGI, HP, SYT, JLX) were defined as *Citrus*-related accessions; *C. trifoliata*, *C. mangshanensis*, *C. linwuensis*, *C. ichangensis*, *C. sinensis*, *C. clementina*, *C. reticulata*, *C. hindsii*, *C. australasica*, *C. medica*, *C. hongheensis*, *C. grandis* (L.) Osbeck cv. Wanbaiyou, *C. maxima* Xipi Majia, *C. grandis* (L.) Osbeck cv. Cupi Majiayou (ZK, MSYG, LW, ZGYCC, SWO, GCF, JZ, GJ, AZM, RL, HH, HWB, XGF, CMJ) were defined as *Citrus* accessions.

PCoA was performed using gene counts of each Gene Ontology (GO) and Kyoto Encyclopedia of Genes and Genomes (KEGG) identifier. Notably, the distribution of gene counts associated with structurally intact TEs differed from those associated with structurally fragmented TEs. For genes overlapping with intact TEs, PCoA based on Bray-Curtis distance demonstrated significant differences between the *Citrus* and *Citrus*-related accessions in both the KEGG and GO identifiers (Adonis test *p* = 0.001, Figure 5a; Adonis test *p* = 0.001, Supplementary Figure S4). In contrast, PCoA for genes overlapping with fragmented TEs showed no significant variation between *Citrus* and *Citrus*-related accessions (Adonis test *p* = 0.065, Figure 5b; Adonis test *p* = 0.022, Supplementary Figure S5). These findings indicate that the functions and pathways of genes overlapping with intact TEs significantly differ between *Citrus* and *Citrus*-related accessions. Collectively, these results suggest that fragmented TEs likely originate from the common ancestors of *Citrus* and *Citrus*-related genera, whereas intact TEs primarily contribute to the recent diversity of *Citrus* and *Citrus*-related accessions.

**Figure 6:**
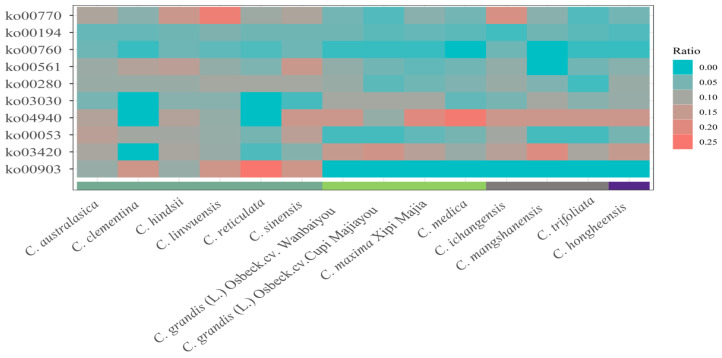
Heatmap for the count ratios of genes in each KEGG pathway. The *p*-value increased from bottom to top; the colored bar at the bottom indicates the level of bitterness.

Functional genes were identified by analyzing the intersection between intact TEs and mRNA, along with their promoters. The number of identified genes in each *Citrus* accession ranged from 1051 to 3879. An unpaired Wilcoxon test was used to compare low-bitterness and high-bitterness samples, with the limonene and pinene degradation (ko00903) pathway ranking first among 445 pathway entries based on *p*-value (Figure 6). Only aldehyde dehydrogenase (ALDH) (K00128), a key enzyme in the glycolysis/gluconeogenesis (ko00010) and ascorbate and aldarate metabolism (ko00053) pathways, was involved in the limonene and pinene degradation pathway in our datasets, suggesting that K00128 genes may play a key role in bitterness.

**Figure 7:**
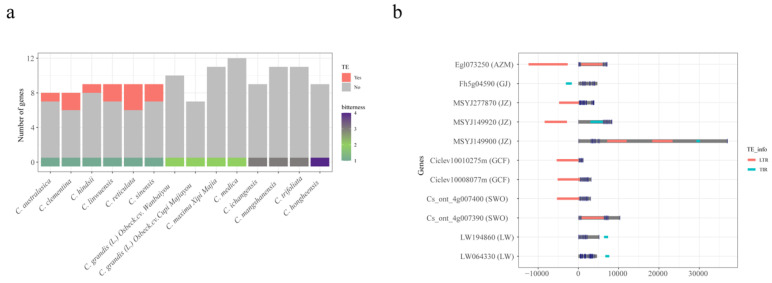
The number of genes in K00128 (**a**) and overview of TE insertions near the target genes. In (**a**), the red box indicates that the genes were overlapped with intact TEs; the colored bar at the bottom indicates the level of bitterness. In (**b**), the *y*-axis represents genes (species); The navy bar represents the CDS region; the red and green bars represent LRT-RTs and TIRs, respectively. *C. australasica*, *C. hindsii*, *C. reticulata*, *C. clementina*, *C. sinensis*, and *C. linwuensis* were abbreviated as AZM, GJ, JZ, GCF, SWO, and LW, respectively.

Based on functional annotation of each genome, a total of 217 candidate genes were identified under the K00128 identifier. A conserved domain search of the National Center for Biotechnology Information (NCBI) database revealed a high confidence level (specific hit) between 206 gene sequences and the Conserved Domain Database (CDD) model. Results showed that 184 of these genes belonged to the aldehyde-alcohol dehydrogenase family (AdhE), including 101 genes containing ALDH active sites (Supplementary Table S4). The intersect screening between intact TEs and the 184 genes and their promoters indicated that 11 genes, all from low-bitterness samples, overlapped with intact TEs (Figure 7a); TIRs and LTR-RTs were involved in these insertion events (Figure 7b). No assembly gaps were found near the 11 genes, indicating their reliability. These findings suggest that the presence and absence of intact TEs near the AdhE superfamily may be highly correlated with the biosynthesis of limonene, a major contributor to bitterness in *Citrus* fruit.

## 3. Discussion

TEs play important roles in the genomes [21,33], contributing to the rapid adaptation and domestication of plants through the introduction of novel traits [10,34]. In the current study, using 20 high-quality assemblies from diverse *Citrus* and *Citrus*-related accessions, we constructed a pan-genome TE library dataset containing 21,680 non-redundant TE sequences, which was then used to reannotate each accession. The re-annotated genomes showed an increase in both the length and number of TEs, along with a decrease in unclassified TEs. Consistent with the known abundance of TEs in the middle of plant chromosomes [10,35], we observed much lower TE insertion proportions at the chromosome ends. *C. grandis* (L.) Osbeck cv. Cupi Majiayou and *C. grandis* (L.) Osbeck cv. Wanbaiyou are two cultivars of the same species, but they show significant differences in TE content. For example, the TE burst time, the half-life ratio, and the number of intact TEs and RT-LTRs of *C. grandis* (L.) Osbeck cv. Cupi Majiayou were 8.09 Mya, 6.28, 15,932, and 2208, respectively. However, these numbers for *C. grandis* (L.) Osbeck cv. Wanbaiyou were 1.51 Mya, 5.15, 14,532, and 1996 (Table 1, Supplementary Tables S1 and S5). In contrast to the genome difference, there are significant differences in botanical traits, such as fruit shape, tree and branch shape, and the color of the juice vesicle, although there is no significant difference in bitterness. Therefore, both genomes were included in our analysis.

Our results further indicated that TE insertions are a major force shaping the genomes of *Citrus* and *Citrus*-related genera. Notably, the number of non-redundant TE sequences, content of different TE types, and half-life of LTR-RTs differed between *Citrus* and *Citrus*-related genera (Supplementary Tables S1 and S5, Table 1). A significant positive correlation was detected between genome size and both the number of consensus TEs and TE proportion in the genome (Figure 3a,b) [13]. Additionally, significant differences in the copy number and size of TE families between *Citrus* and *Citrus*-related genera were observed (Figure 4a,b). The early-diverging *Citrus* species (*C. ichangensis*, *C. mangshanensis*, and *C. trifoliata*) originated around 10 Mya, which is a short evolutionary period for sequence divergence for a perennial species with a long intergenerational period, resulting in highly similar functional genes between *Citrus* and *Citrus*-related genera [13]. These findings suggest that functional genes are not the primary cause of genome differences. Furthermore, the large half-life rate of TEs in the 20 *Citrus* and *Citrus*-related genera indicated a lower rate of LTR-RT DNA removal (Table 1, median 6.92 million years), implying that the removal of LTR-RTs was not the main cause of genome variation. Collectively, TE insertions may contribute significantly to interspecific and intraspecific variability among *Citrus* and *Citrus*-related genera.

TEs exist as both structurally intact and fragmented sequences in eukaryotic genomes [32,36]. Fragmented TEs result from mutations of intact TEs [2,37]. If insertion and deletion rates of TE superfamilies are constant, it is likely that genomic insertion of intact TEs occurred more recently than that of fragmented TEs [38]. In this study, GO and KEGG identifiers were used to represent gene functions and pathways, respectively. Genes overlapping with TEs were involved in a wide variety of biological functions, including negative regulation of different metabolic and biosynthetic processes, glucan metabolic and biosynthetic processes, and cellular responses, suggesting that TEs have been pervasively co-opted to modify host genes in *Citrus* and *Citrus*-related genera. The high similarity in the functions of genes overlapping with fragmented TEs between *Citrus* and *Citrus*-related genera suggests that these fragmented TEs originated from their common ancestors or that TEs tend to insert around specific types of genes. Additionally, a significant difference was found between *Citrus* and *Citrus*-related accessions in the functions of genes overlapping with intact TEs, illustrating that the insertion of LTR-RTs exhibits non-genetic preference. Thus, we propose that fragmented TEs are ancestral to *Citrus* and *Citrus*-related genera, and after the divergence of *Citrus*, TEs continued to insert into genomes, shaping their genomic organization.

Limonene and limonoids are primarily responsible for bitterness in *Citrus* [39]. We observed the presence and absence of TE insertions in several genes of the AdhE superfamily between low-bitterness and high-bitterness *Citrus* varieties (Figure 6 and Figure 7a). The AdhE superfamily consists of two catalytic domains of ALDH and alcohol dehydrogenase (ADH) active sites, which are involved in limonene degradation and glycolysis/gluconeogenesis [40]. In this study, 10 out of 11 insertions occurred within genes containing ALDH active sites. Notably, these TE insertions were not located in the coding sequence (CDS) regions but rather at the intron regions and upstream or downstream of the CDS regions (Figure 7b). Overall, we propose that the insertions of intact TEs around AdhE have significant implications for *Citrus* bitterness.

## 4. Materials and Methods

### 4.1. Genome Dataset Collection

The genomes of 20 *Citrus* and *Citrus*-related genera were downloaded from the Citrus Pan-genome to Breeding Database (CPBD, http://citrus.hzau.edu.cn) [31] for the detection, annotation, and analysis of TEs. Genome assembly information is provided in Table 1. The accessions *A. marmelos*, *A. buxifolia*, *C. gilletiana*, *C. lansium*, *L. scandens*, and *M. paniculata* (AEG, HKC, CGI, HP, SYT, JLX) were classified as *Citrus*-related, and *C. trifoliata*, *C. mangshanensis*, *C. linwuensis*, *C. ichangensis*, *C. sinensis*, *C. clementina*, *C. reticulata*, *C. hindsii*, *C. australasica*, *C. medica*, *C. hongheensis*, *C. grandis* (L.) Osbeck cv. Wanbaiyou, *C. maxima* Xipi Majia, *C. grandis* (L.) Osbeck cv. Cupi Majiayou (ZK, MSYG, LW, ZGYCC, SWO, GCF, JZ, GJ, AZM, RL, HH, HWB, XGF, CMJ) were defined as *Citrus* accessions. Fully ripened fruits were collected for bitterness assessment. Juice sacs and segment membranes of each sample were detached and assessed by 5–10 experienced human tasters. A score of 1 indicated no bitterness, while a score of 4 represented the highest level of bitterness.

### 4.2. Construction of the Pan-Genome TE Library

The TEs of each accession were identified using the extensive *de-novo* TE annotator (EDTA, v2.0.1), a pipeline designed for comprehensive and high-quality TE annotation [32]. A *Citrus* pan-genome TE library was generated by combining the non-redundant TEs of the 20 *Citrus* and *Citrus*-related genera. Initially, structurally intact TEs were identified in each TE library, with sequences shorter than 80 bp discarded. Each TE library was then added in random order to construct a pan-genome TE library. Redundant and highly similar sequences were removed using the make_panTElib.pl, script in the EDTA package with parameters ‘-miniden 80 -mincov 80′. Finally, the non-redundant pan-genome TE library was re-classified using DeepTE [41].

### 4.3. Genome Reannotation with the Pan-Genome TE Library

The pan-genome TE library was subsequently used to re-annotate whole-genome TEs in the 20 assemblies using EDTA. Intact LTR-RT information was extracted from the EDTA results, and insertion times were calculated using the formula T = K/2µ = (1 − identity)/2µ, where identity represents the identity of intact LTR-RTs, and µ is the synonymous substitutions per site per year for *Citrus* and *Citrus*-related genera (1.34 × 10^−9^). According to Huang et al., insertion times were categorized into three periods: past insertion (>10 Mya), early insertion (6.84 < insertion time ≤ 10 Mya), and recent insertion (≤ 6.84 Mya) [13]. We further estimated the half-life rate for the removal of full-length LTR-RTs from the genome. Assuming a constant rate of LTR-RT removal, the distribution of insertion times can be modeled by an exponential function with a constant half-life rate. To estimate the half-life rate of LTR-RTs, exponential decay functions were fitted to the age distributions using the fitdistr function of R/MASS (v7.3-60), with the half-lives calculated according to Chu et al. [42].

The copy number and physical size of each TE family in the re-annotated dataset were analyzed using PCoA. Copy number and TE family size were assigned a value of 0 in genomes where TEs were absent. Sample diversity was calculated based on Bray-Curtis dissimilarity and PCoA, then visualized using the ggplot2 and vegan packages in R (v4.0.5) [43]. In addition, permutational analysis of molecular variance (Adonis or PERMANOVA, number of permutations = 999) was conducted to test for significant differences between *Citrus* and *Citrus*-related accessions.

### 4.4. Intact TEs and Citrus Bitterness

Genome-wide functional annotation was performed using eggnog-mapper (http://eggnog-mapper.embl.de/, accessed on 5 October 2023). Overlaps between TEs and regions 3000 bp upstream and downstream of genes were screened using bedtools (v2.25) [44]. GO and KEGG identifiers of the overlapped genes were then extracted. The gene count for each identifier was normalized by dividing the number of genes overlapping with TEs by the total number of genes in each identifier for each genome. PCoA and Adonis analyses were performed as described above.

The mRNA and 3000 bp upstream promoter regions of 14 *Citrus* genomes were defined as the target regions. Potential candidate genes were identified by scanning the intersection between intact TEs and the target region using bedtools (v2.25). The gene count for each KEGG identifier was normalized by dividing the number of genes overlapping with TEs by the total number of genes in each identifier for each genome. The unpaired Wilcoxon test was used to compare low-bitterness (bitterness level of 1) and high-bitterness (bitterness level of 2–4) accessions. Conserved domains in protein sequences were identified using the conserved domains and protein classification tool of the NCBI [45].

## 5. Conclusions

In this study, we constructed a pan-genome TE library using 20 published genomes of *Citrus* and *Citrus*-related accessions to uniformly re-annotate TEs across all genomes. Comparative analysis of TEs in the 20 genomes helped clarify the composition of TEs in each genome and their roles in genome evolution and function. While TEs were identified as critical determinants of genome size, their removal had no obvious effect on genome size. Moreover, the presence and absence of intact TEs near the AdhE superfamily were closely related to the bitterness trait of *Citrus* genera, providing a theoretical basis for future *Citrus* cultivation.

## Figures and Tables

**Table 1:** Essential information, burst time, and half-life ratio of LTR-RTs in the 20 genomes.

Latin Name	Code Name	Bitterness	Version	Assembly Level	Burst Time	Half-Life Ratio
*A. marmelos*	AEG		v1	Contig	11.86	9.51
*C. australasica*	AZM	1	v1	Contig	7.15	7.97
*C. gilletiana*	CGI		v1	Contig	5.19	7.11
*C. grandis* (L.) Osbeck. cv. Cupi Majiayou	CMJ	2	v1	Chromosome	8.09	6.28
*C. clementina*	GCF	1	v1	Scaffold	1.90	5.59
*C. hindsii*	GJ	1	v1	Chromosome	0.99	4.20
*C. hongheensis*	HH	4	v1	Contig	5.43	7.56
*A. buxfoliata*	HKC		v2	Chromosome	4.09	6.59
*C. lansium*	HP	1	v1	Contig	2.48	8.18
*C. grandis* (L.) Osbeck. cv. Wanbaiyou	HWB	2	v1	Chromosome	1.51	5.15
*M. paniculata*	JLX		v1	Scaffold	2.55	7.17
*C. reticulata*	JZ	1	v1	Scaffold	7.01	8.35
*C. linwuensis*	LW	1	v1	Contig	3.79	6.16
*C. mangshanensis*	MSYG	3	v1	Contig	3.21	6.19
*C. medica*	RL	2	v1	Scaffold	2.44	7.84
*C. sinensis*	SWO	1	v3	Chromosome	4.68	5.76
*L. scandens*	SYT		v1	Contig	8.03	8.37
*C. maxima* Xipi Majia	XGF	2	v1	Contig	8.54	7.28
*C. ichangensis*	ZGYCC	3	v2	Contig	4.07	5.57
*C. trifoliata*	ZK	3	v1	Chromosome	1.66	6.71

Bitterness: the sample that did not taste bitter was recorded as 1; the bitterness from the lowest to the highest was recorded as 2, 3, and 4; the blank represented no-record samples.

**Table 2:** Summary of the Citrus pan-genome TE library.

Class	Order	Superfamily	Count
Class I	DIRS	*DIRS*	2
Class I	LINE	*I*	4
Class I	LINE	*L1*	36
Class I	LINE	unknown	196
Class I	LTR	*Copia*	4072
Class I	LTR	*Gypsy*	3427
Class I	LTR	Retrovirus	54
Class I	LTR	unknown	116
Class I	PLE	*Penelope*	38
Class I	nonLTR	unknown	10
Class I	SINE	SINE	57
Class II	Helitron	*Helitron*	125
Class II	Unknown		724
Class II	TIR	*CACTA*	1170
Class II	TIR	*Mutator*	3258
Class II	TIR	*PIF_Harbinger*	753
Class II	TIR	*Tc1_Mariner*	1382
Class II	TIR	*Transib*	2
Class II	TIR	*hAT*	4866
Class II	TIR	unknown	1388

## Data Availability

Data are contained within the article.

## Supplementary Materials

The following supporting information can be downloaded at: https://www.mdpi.com/article/10.3390/plants13172462/s1, Table S1. Summary of the TE Annotations; Table S2. The copy number difference of TE families between Citrus and Citrus-related genera; Table S3. The size difference of TE families between Citrus and Citrus-related genera; Table S4. The conserved domains of the genes in K00128; Table S5. Appendix of genome and TE annotation; Figure S1. TEs percentages in the 20 genomes used in this study. The percentages of LTR (Long terminal repeat), TIR (Terminal inverted repeat), and Repeat_region (Unclassified element), Helitron (Helitron DNA transposon), LINE (Long interspersed nuclear element), PLE (Penelope-like element), SINE (Short interspersed nuclear element), and DIR (Dictyostelium intermediate repeat sequence) in genomes were estimated with EDTA; Figure S2. Unclassed TEs (repeat region) in original annotated and re-annotated genomes; Figure S3. Distribution of TEs in each chromosome. a-i indicted chromosome 1—chromosome 9. GJ (*C. hindsii*), SWO (*C. sinensis*), CMJ (*C. grandis* (L.) Osbeck cv. Cupi Majiayou), HWB (*C. grandis* (L.) Osbeck cv. Wanbaiyou), ZK (*C. trifoliata*), HKC (*A. buxfoliata*); Figure S4. PCoA plots for GO identifier distribution of genes that overlapped with intact TEs between the *Citrus* and *Citrus*-related accessions. *A. marmelos*, *A. buxfoliata*, *C. gilletiana*, *C. lansium*, *L. scandens*, *M. paniculate* (AEG, HKC, CGI, HP, SYT, JLX) were defined as *Citrus*-related accessions; *C. trifoliata*, *C. mangshanensis*, *C. linwuensis*, *C. ichangensis*, *C. sinensis*, *C. clementina*, *C. reticulata*, *C. hindsii*, *C. australasica*, *C. medica*, *C. hongheensis*, *C. grandis* (L.) Osbeck cv. Wanbaiyou, *C. maxima* Xipi Majia, *C. grandis* (L.) Osbeck cv. Cupi Majiayou (ZK, MSYG, LW, ZGYCC, SWO, GCF, JZ, GJ, AZM, RL, HH, HWB, XGF, CMJ) were defined as *Citrus* accessions; Figure S5. PCoA plots for GO identifier distribution of genes that overlapped with fragmented TEs between the *Citrus* and *Citrus*-related accessions. *A. marmelos*, *A. buxfoliata*, *C. gilletiana*, *C. lansium*, *L. scandens*, *M. paniculate* (AEG, HKC, CGI, HP, SYT, JLX) were defined as *Citrus*-related accessions; *C. trifoliata*, *C. mangshanensis*, *C. linwuensis*, *C. ichangensis*, *C. sinensis*, *C. clementina*, *C. reticulata*, *C. hindsii*, *C. australasica*, *C. medica*, *C. hongheensis*, *C. grandis* (L.) Osbeck cv. Wanbaiyou, *C. maxima* Xipi Majia, *C. grandis* (L.) Osbeck cv. Cupi Majiayou (ZK, MSYG, LW, ZGYCC, SWO, GCF, JZ, GJ, AZM, RL, HH, HWB, XGF, CMJ) were defined as *Citrus* accessions.
