# Supplementary material for: Comparative Analysis of Transposable Elements in the Genomes of Citrus and Citrus-Related Genera
Source: Plants (Basel). 2024 Sep 3;13(17):2462. doi: 10.3390/plants13172462 (PMC11397423; doi:10.3390/plants13172462)
Supplement: Supplementary file 1 [file plants-13-02462-s001.zip › Supplementary Figures.pdf]

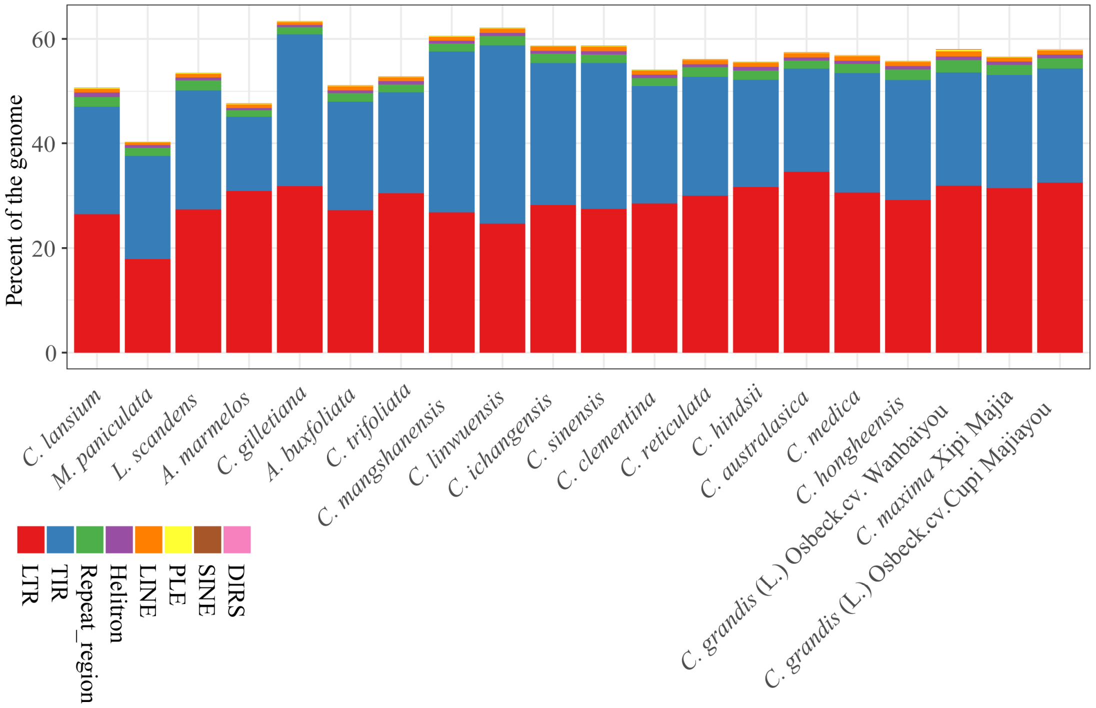

Supplementary Figure S1. TEs percentages in the 20 genomes used in this study. The percentages of LTR (Long terminal repeat), TIR (Terminal inverted repeat), and Repeat_region (Unclassified element), Helitron (Helitron DNA transposon), LINE (Long interspersed nuclear element), PLE (Penelope-like element), SINE (Short interspersed nuclear element), and DIR (Dictyostelium intermediate repeat sequence) in genomes were estimated with EDTA.

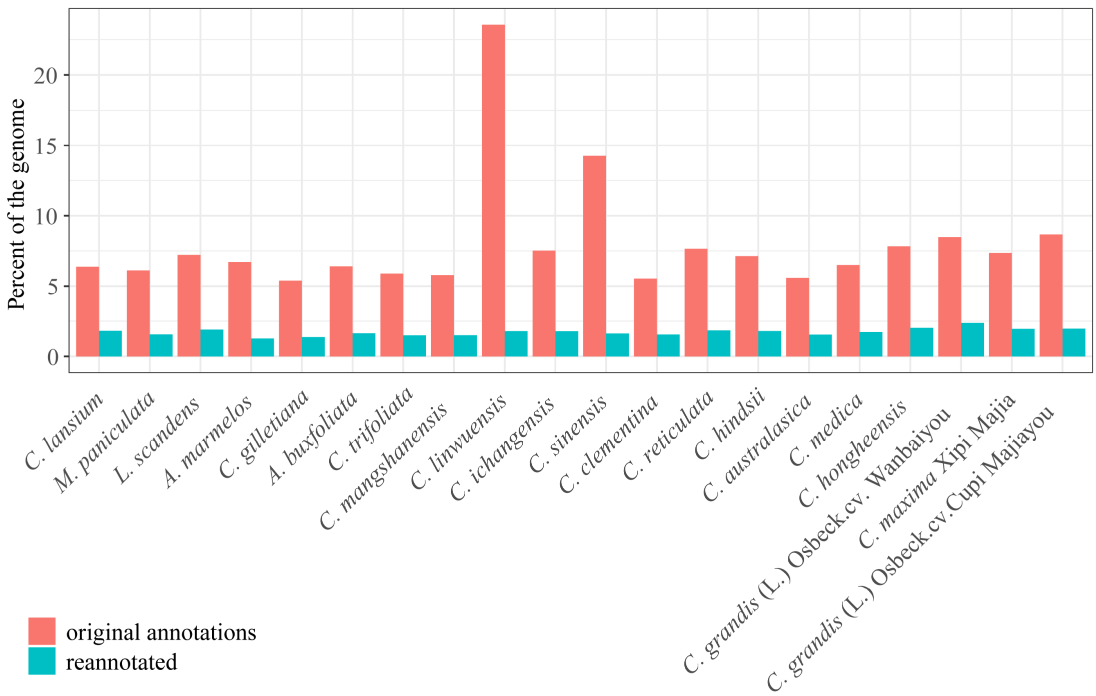

Supplementary Figure S2. Unclassed TEs (repeat region) in original annotated and re-annotated genomes.

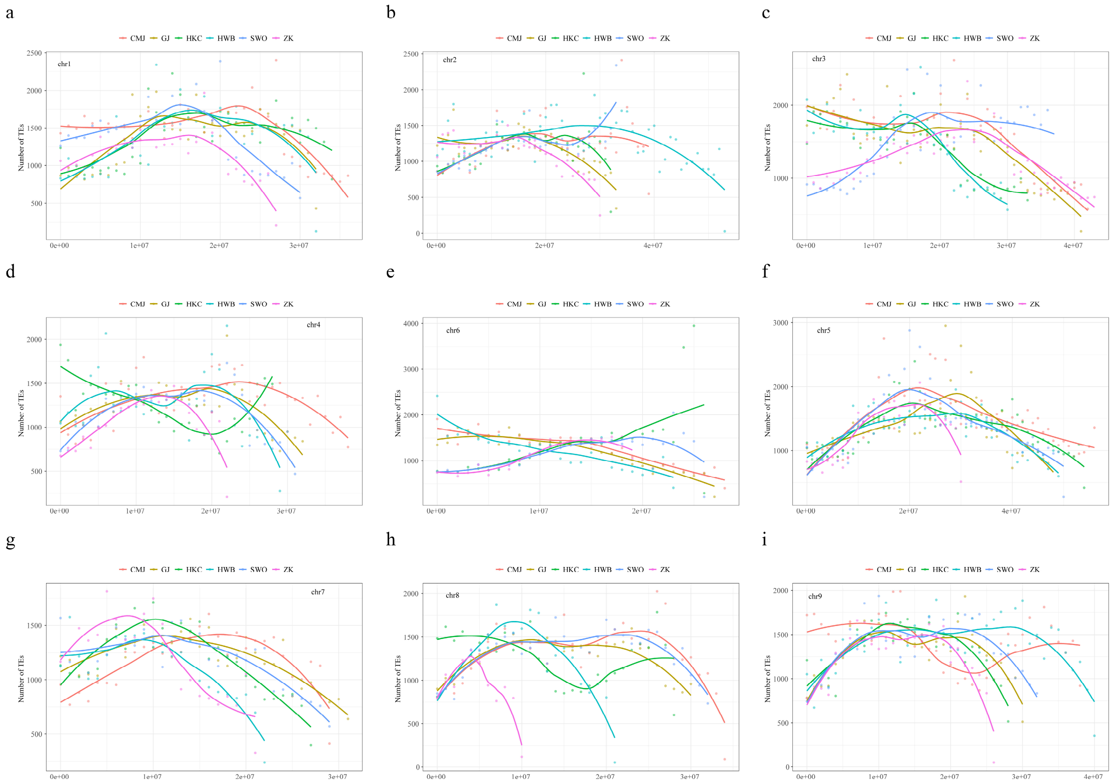

Supplementary Figure S3. Distribution of TEs in each chromosome. a-i indicted chromosome 1 - chromosome 9. GJ (*C. hindsii*), SWO (*C. sinensis*), CMJ (*C. grandis* (*L.*) *Osbeck. cv. Cupi Majiayou*), HWB (*C. grandis* (*L.*) *Osbeck.cv. Wanbaiyou*), ZK (*C. trifoliata*), HKC (*A. buxfoliata*).

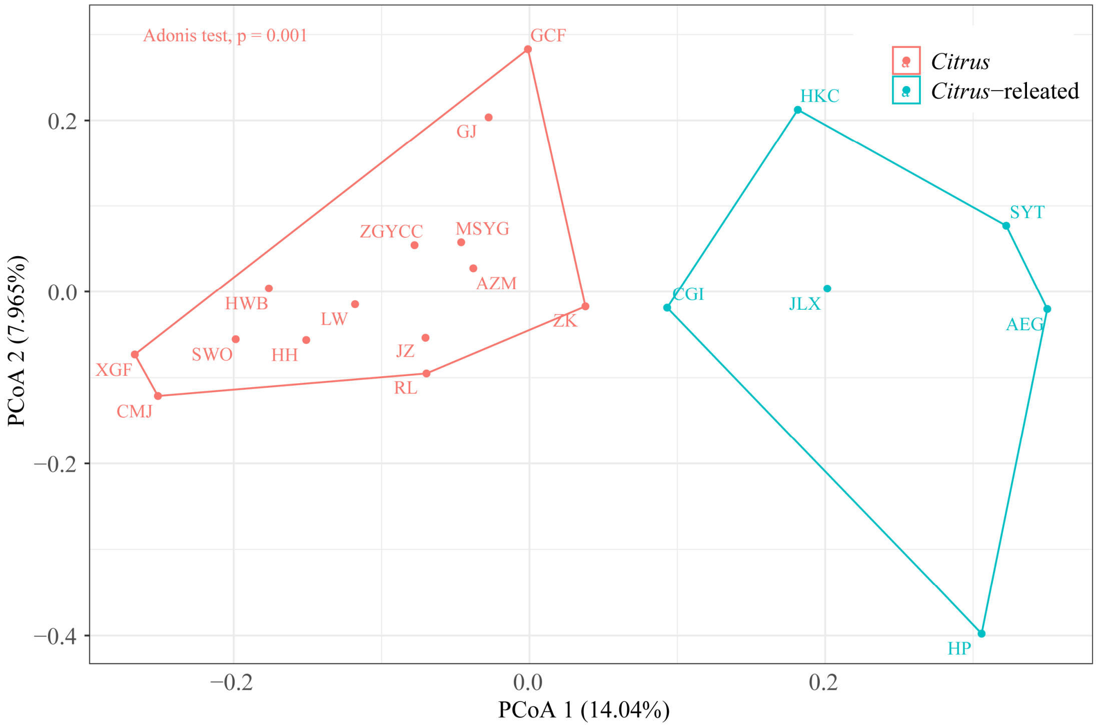

Supplementary Figure S4. PCoA plots for GO identifier distribution of genes that overlapped with intact TEs between the *Citrus* and *Citrus*-related accessions. *A. marmelos, A. buxfoliata, C. gilletiana, C. lansium, L. scandens, M. paniculate* (AEG, HKC, CGI, HP, SYT, JLX) were defined as *Citrus*-related accessions; *C. trifoliata*, *C. mangshanensis*, *C. linwuensis*, *C. ichangensis*, *C. sinensis*, *C. clementina*, *C. reticulata*, *C. hindsii*, *C. australasica*, *C. medica*, *C. hongheensis*, *C. grandis* (L.) Osbeck.cv. Wanbaiyou, *C. maxima* Xipi Majia, *C. grandis* (L.) Osbeck.cv.Cupi Majiayou (ZK, MSYG, LW, ZGYCC, SWO, GCF, JZ, GJ, AZM, RL, HH, HWB, XGF, CMJ) were defined as *Citrus* accessions.

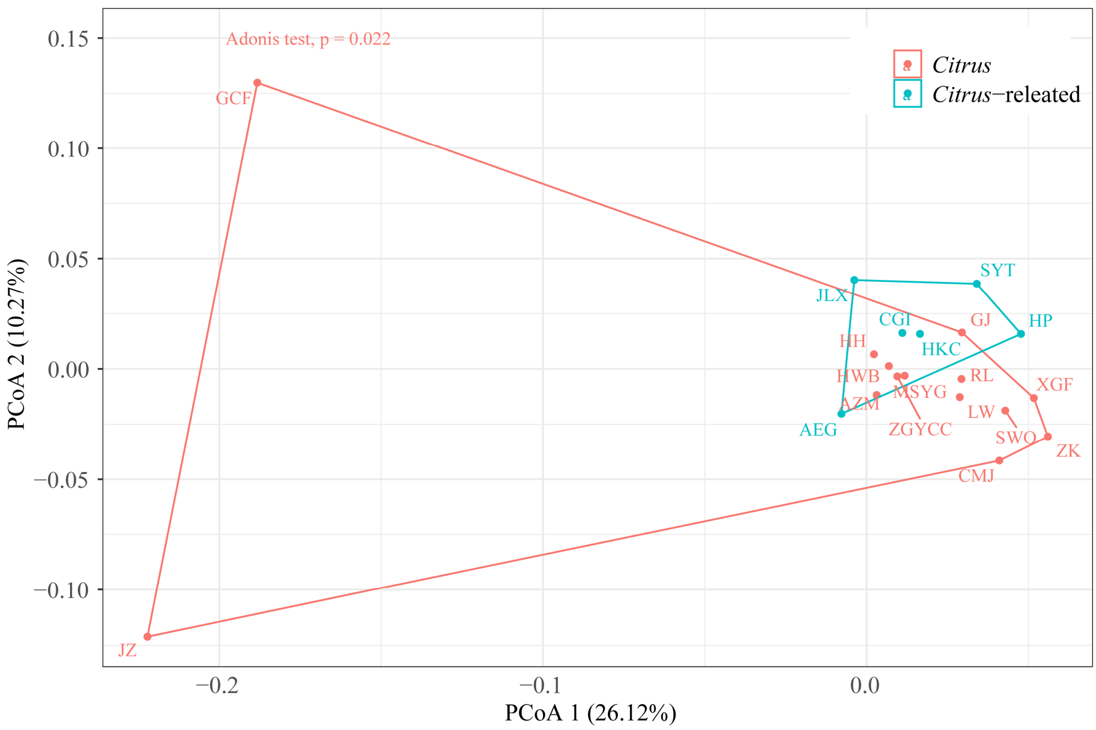

Supplementary Figure S5. PCoA plots for GO identifier distribution of genes that overlapped with fragmented TEs between the *Citrus* and *Citrus*-related accessions. *A. marmelos, A. buxfoliata, C. gilletiana, C. lansium, L. scandens, M. paniculate* (AEG, HKC, CGI, HP, SYT, JLX) were defined as *Citrus*-related accessions; *C. trifoliata*, *C. mangshanensis*, *C. linwuensis*, *C. ichangensis*, *C. sinensis*, *C. clementina*, *C. reticulata*, *C. hindsii*, *C. australasica*, *C. medica*, *C. hongheensis*, *C. grandis* (L.) Osbeck.cv. Wanbaiyou, *C. maxima* Xipi Majia, *C. grandis* (L.) Osbeck.cv.Cupi Majiayou (ZK, MSYG, LW, ZGYCC, SWO, GCF, JZ, GJ, AZM, RL, HH, HWB, XGF, CMJ) were defined as *Citrus* accessions
